# The Causation of Epidemics

**Published:** 1909-07-03

**Authors:** 


### THE CAUSATION OF EPIDEMICS.

At a recent meeting of the Epidemiological Section of the Royal Society of Medicine, a paper by Dr. John Brownlee on "Certain Considerations on the Causation and Course of Epidemics" raised an interesting question on which medical opinion seems destined to be divided. The issue received emphasis from another paper read the same evening by Dr. M. Greenwood, of the London Hospital Statistical Laboratory on "The Problem of Marital Infection in Pulmonary Tuberculosis." The titles of the two papers do not suggest an identical problem, but essentially they raise the same question: Does infectivity depend upon the distribution of the infective agent, or upon the susceptibility of the persons exposed? Put thus, it is obvious that the answer is—upon both. Nevertheless, there is a real issue, and although one may cavil at the width of the generalisations implied they serve to mark two distinct schools of medical thought.

If, on the one hand, it be considered how much energy is devoted to the destruction, or to limiting the diffusion, of infective material, it will be seen that underlying this action is the view, perhaps somewhat loosely held, that in this method is to be found the most potent weapon of checking the spread of disease. On the other hand, there are those who, like Dr. Archdale Reid or Professor Karl Pearson, hold that in the varying degree of susceptibility to certain infections is to be found the main condition of epi- or en-demicity. At the risk of unwillingly misrepresenting them it is necessary, in order to understand the issue, to put the two views in opposition.

Why is it, asks Professor Karl Pearson, that the sons of tuberculous fathers show an incidence of tubercular infection which we may represent as .6, while the wives of tuberculous husbands have an incidence which may be represented as .3? The intimacy of a relationship capable of conveying infection is very much greater in the case of husband and wife than is that of father and son. But in the case of a son we have presumably reproduced the tissue vulnerability which in the father was a main determinant of the attack; while in a wife, notwithstanding an unquestionably more intimate exposure, we have the chance insusceptibility which a random sample of the female population identically exposed would show.

We are stating the question somewhat figuratively in our own way, and trust we are doing no injustice to those who find in it support for the view that there is in the population generally a wide variation in degree of susceptibility to disease sufficient to account for some of the chief features of its distribution. It is urged in opposition to this view that the data on which it is based are insufficient or unreliable, and that were there recorded observations sufficiently exact and comprehensive they would show that the distribution of tuberculosis, for instance, coincides with the opportunities afforded to those, who actually, become infected, of receiving a sufficient dose of the bacilli.

At the present time preventive measures are based mainly upon the supposition that susceptibility to tuberculosis is a common inheritance, and that the decline in the disease is due to the lessened chances of exposure to critical infection. According to Dr. Newsholme, increased institutional segregation of the tuberculous has been chiefly accountable for this diminished exposure; and those who think with him look for further reduction of tubercular mortality by controlling the channels of infection, guarding against personal infection by proper regulation of the life of tuberculous persons, and eradicating the disease from domestic animals, more especially those used for the food of man. If tuberculous meat and milk and sputum are the means of conveying the disease, what more rational pursuit than to eradicate such products? It is urged, on the other hand, that decline in the phthisical death-rate cannot be due to a lessened prevalence of infective material or to diminished opportunities of exposure to infection. Urbanisation has led to so much more intimate intercourse of all classes as more than to neutralise any problematical benefit ascribed to workhouse infirmary treatment of advanced phthisis. It is extremely doubtful whether tubercle-infected food is not now as widely consumed as at any period during the decline in tubercular mortality.

The chances of exposure in fact are such to-day that, granted a high degree of susceptibility, it is morally certain that the disease will be contracted. The excessive incidence of the disease, generation after generation, upon certain families, which has for long been observed, is still a phenomenon of contemporary tuberculosis. Its epidemic features remain the same, but there is a lessened volume of the disease. If the potency of infectivity has declined because of more limited dissemination of the infective material why do those hereditarily highly susceptible to tubercle still show the evidence of this in an excessive mortality from the disease?

The real and important element in its causation, it is argued upon these grounds, is the variation in susceptibility, a variation accounted for partly upon grounds of heredity and partly upon grounds of permanent environment. The resistance to tubercle has been reinforced by the better feeding, better housing, and improved hygiene generally which have advanced *pari passu* with the decline of the disease; and in this lessened vulnerability to a specific infection, is to be found (in the case of tubercle at all events) the main cause of its decline.

Dr. Brownlee's paper states that an epidemic dies out because of the exhaustion of susceptible persons or the loss of infectivity of an organism. In certain epidemics such as influenza, where the diffusion of the infection is such that practically everyone is exposed to the infectious material, such an alternative seems probable; but an epidemic of typhoid fever, for instance, is nowadays usually terminated because the greater part of the population is protected from exposure to the typhoid bacillus. The proportion of persons insusceptible to this infection is probably small, but comparatively few people take the disease because the main channels of its convection have been closed. Smallpox, on the other hand, has been practically abolished in Europe and America by immunising the otherwise susceptible populations to the infection. Secure from intelligent interference, its natural epidemic curve would be comparable with that of measles and influenza to-day.

These diseases, analogous in their high degree of infectivity, are yet demonstrably modified by variations of susceptibility in those exposed. Disturbance arising from exhaustion of susceptible persons exposed, from interference profoundly modifying the exposure risk for large sections of the community, from accidental variation in the degree of susceptibility, and from the intimacy of the exposure—must necessarily reflect itself in a curve which represents the epidemic reaction to the interplay of all these and other unspecified factors. To speak of the cause of epidemics is thus at the best to refer to a complex of analysed conditions whose correlation is only dimly apprehended.

